# Concomitant occurrence of clinoid and cavernous segment aneurysms complicated with carotid cavernous fistula: A case report

**DOI:** 10.1097/MD.0000000000018184

**Published:** 2019-11-27

**Authors:** Wang Ting, Seidu A. Richard, Zhang Changwei, Wang Chaohua, Xie Xiaodong

**Affiliations:** a Department of Neurosurgery, West China Hospital, Sichuan University, Chengdu, PR China; b Department of Medicine, Princefield University, Ho-Volta Region, Ghana.

**Keywords:** CCA, CCF, CSA, ICA, PLED

## Abstract

**Rationale:**

Dual aneurysms arising from the internal cerotic artery (ICA) is a very rare occurrence. Clinoid segment aneurysms (CSAs) are often seen at the carotid dural rings while cavernous carotid aneurysms (CCAs) are often a direct communication between the ICA and the cavernous sinus (CS). We present a case of complex concomitant occurrence of a CSA and a CCA complicated with delay aneurysmal rupture (DAR) resulting in carotid cavernous fistula (CCF) after our initial treatment of the patient with pipeline embolization devices (PLEDs)

**Patient Concerns:**

We present a 64-year old female who we admitted at our institution due to one-year history of double vision. Neurological examinations were unremarkable.

**Diagnosis:**

Magnetic resonance imaging (MRI) and computer tomography (CT)-scan revealed dual aneurysms on the ICA. Digital subtracting angiogram (DSA) confirmed a small CSA and a large CCA on the right ICA.

**Interventions:**

We treated both aneurysms with PLED and subsequently observed DAR of CCA as a complication.

**Outcomes:**

We successfully occluded the fistula with ONYX (ev3, Irvine, CA) via the trans-venous approach.

**Lesions:**

PLED was the best endovascular treatment option though DAR was inevitable. Although the trans-arterial approach may be the gold standard for the managing of CCF, the complex nature of our case made us opt for trans-venous approach. The trans-venous route is very appropriate for fistulas with complex parent arteries.

## 1 Introduction

Multiple aneurysms arising from the internal cerotic artery (ICA) is a very rare occurrence. Anatomically, the clinoidal segment is the portion of the ICA that is located between the cavernous sinus (CS) and the subarachnoid space.^[1,2]^ Clinoid segment aneurysms (CSAs) usually originates from the carotid dural rings.^[2,3]^ CSAs are often seen between the proximal and distal ends of the ring.^[3,4]^ The complex landmarks of CSAs often make their diagnosis and treatment difficult.^[4,5]^ They often carry high risks of rupture because they occupy a tiny segment of the ICA.^[1,4]^

Cavernous carotid aneurysms (CCAs) are often a direct communication between the ICA and the CS.^[6,7]^ CCAs accounts for about 2% to 9% of all aneurysms in the brain.^[8,9]^ These lesions often arise as a result of traumatic events although cases of idiopathic, infectious as well as iatrogenic have been reported.^[9,10]^ They are usually asymptomatic during their initial development but become symptomatic when they grow into large and giant sizes.^[11]^ Their mass effects on adjacent cranial nerve often lead to diplopia, ophthalmoplegia, ptosis, pain as well as paresthesia.^[9]^ CCAs often have a low risk of causing major deformities as well as death except when they result into a fistula.^[12]^

A carotid cavernous fistula (CCF) is an abnormal communication between the ICA and the CS as a result of trauma or rupture of an antecedent carotid segment aneurysm.^[13,14]^ Studies have proven that, flow diverting devices (FDDs) treatment option often result in aneurysm rupture or parent vessel injury resulting in high flow shunts between the ICA and the CS, leading to CCF.^[15,16]^ This often present as subarachnoid hemorrhage (SAH).^[16,17]^ We present a complex occurrence of clinoid and cavernous segment aneurysms complicated with CCF as a result of delay aneurysm rapture (DAR) after our initial treating with pipeline embolization device (PLED).

## 2 Case report

We present a 64-year old female who we admitted at our institution due to one-year history of double vision. General physical examination did not yield much during our assessment. Cranial nerves examination was unremarkable. Magnetic resonance imaging (MRI) and Computer tomography (CT)-scan revealed dual aneurysms on the ICA. A confirmatory digital subtracting angiogram (DSA) revealed a small CSA and a large cavernous segment aneurysm or CCA on the right ICA **(**Fig. 1  A). The endovascular curative procedure was done under general anesthesia with FFDs. We successfully implanted PLED (ev3, Irvine, CA) across both aneurysms. We preferred PLED because of the location and number of the aneurysms. Five days prior to the procedure the patient was started on aspirin 75 mg and clopidogrel 75 mg.

**Figure 1:**
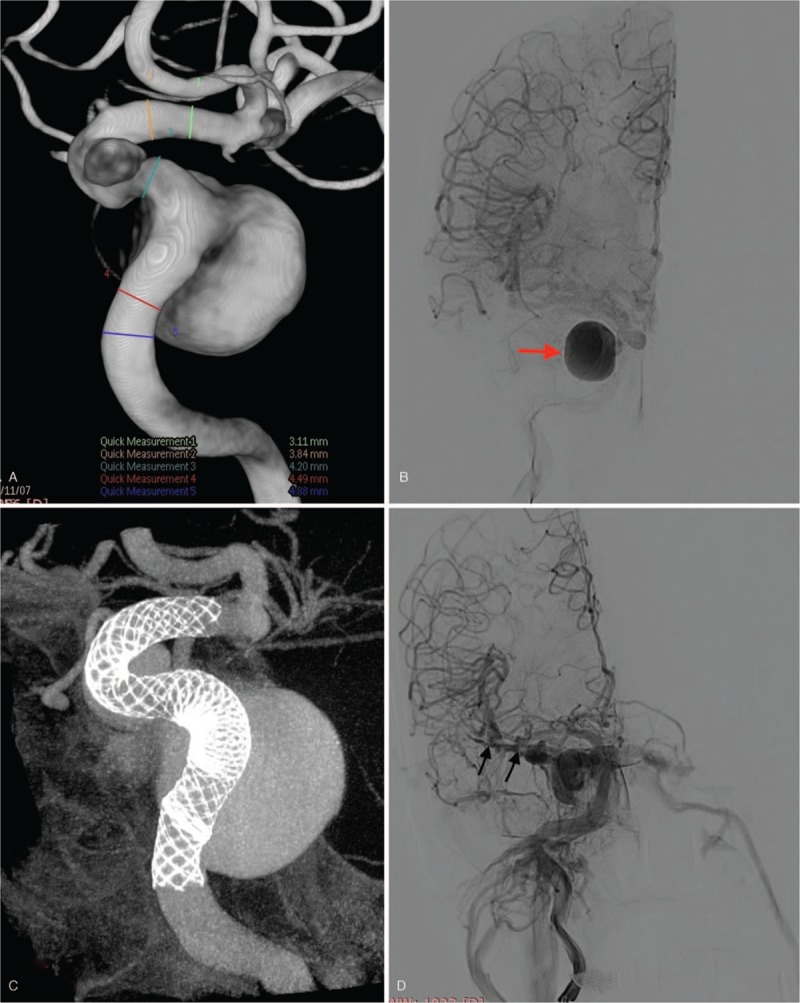
A: 3D reconstruction disclosed a small clinoidal segment aneurysm and a large cavernous segment aneurysm of the right internal carotid artery (ICA). B: frontal view: post-operative angiogram showed obvious contrast retention in the large aneurysm sac (red arrow), while not in the small. C: The Vaso-CT displayed satisfactory apposition of the stent which bridged the two aneurysmal necks completely. D: Frontal view: sphenoparietal sinus (SS) or cortical vein drainage (black arrow) of the CCF following PLED. E: Lateral view: superior ophthalmic vein (SOV, white arrow), pterygoid plexus (PP, white asterisk), inferior petrosal sinuses (IPS, black arrow) and superior petrosal sinuses (SPS, red arrow). F: The microcatheter tip locates in the origin of right SOV (white arrow). G: Frontal view: Showing a little SS (white arrow) and PP drainage (white asterisk) after the second procedure. H: Lateral view: Showing a little SS (white arrow) and PP drainage (white asterisk) after the second procedure. I: Frontal view of DSA during 4-month follow-up showing complete occlusion of the CCF. J: Lateral view of DSA during 4-month follow-up showing complete occlusion of the CCF.

**Figure 1 (Continued):**
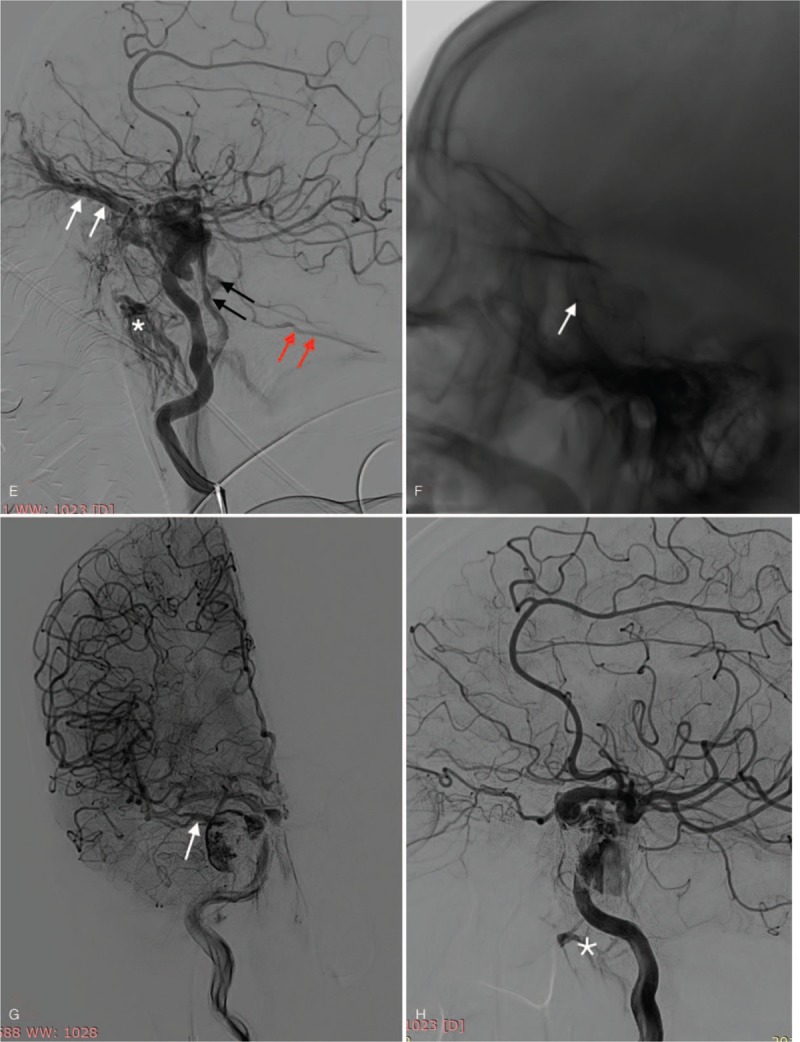
A: 3D reconstruction disclosed a small clinoidal segment aneurysm and a large cavernous segment aneurysm of the right internal carotid artery (ICA). B: frontal view: post-operative angiogram showed obvious contrast retention in the large aneurysm sac (red arrow), while not in the small. C: The Vaso-CT displayed satisfactory apposition of the stent which bridged the two aneurysmal necks completely. D: Frontal view: sphenoparietal sinus (SS) or cortical vein drainage (black arrow) of the CCF following PLED. E: Lateral view: superior ophthalmic vein (SOV, white arrow), pterygoid plexus (PP, white asterisk), inferior petrosal sinuses (IPS, black arrow) and superior petrosal sinuses (SPS, red arrow). F: The microcatheter tip locates in the origin of right SOV (white arrow). G: Frontal view: Showing a little SS (white arrow) and PP drainage (white asterisk) after the second procedure. H: Lateral view: Showing a little SS (white arrow) and PP drainage (white asterisk) after the second procedure. I: Frontal view of DSA during 4-month follow-up showing complete occlusion of the CCF. J: Lateral view of DSA during 4-month follow-up showing complete occlusion of the CCF.

**Figure 1 (Continued):**
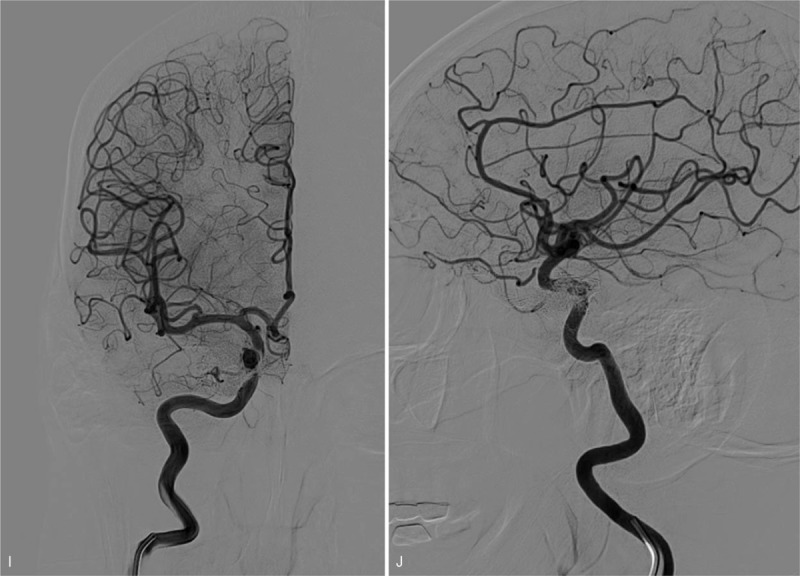
A: 3D reconstruction disclosed a small clinoidal segment aneurysm and a large cavernous segment aneurysm of the right internal carotid artery (ICA). B: frontal view: post-operative angiogram showed obvious contrast retention in the large aneurysm sac (red arrow), while not in the small. C: The Vaso-CT displayed satisfactory apposition of the stent which bridged the two aneurysmal necks completely. D: Frontal view: sphenoparietal sinus (SS) or cortical vein drainage (black arrow) of the CCF following PLED. E: Lateral view: superior ophthalmic vein (SOV, white arrow), pterygoid plexus (PP, white asterisk), inferior petrosal sinuses (IPS, black arrow) and superior petrosal sinuses (SPS, red arrow). F: The microcatheter tip locates in the origin of right SOV (white arrow). G: Frontal view: Showing a little SS (white arrow) and PP drainage (white asterisk) after the second procedure. H: Lateral view: Showing a little SS (white arrow) and PP drainage (white asterisk) after the second procedure. I: Frontal view of DSA during 4-month follow-up showing complete occlusion of the CCF. J: Lateral view of DSA during 4-month follow-up showing complete occlusion of the CCF.

We used a triaxial system comprising of a 7 French 90 cm cook long sheath, a 5 French 115 cm navien, and a marksman to provide support for the PLED implantation. A 4.5 mm × 35 mm PLED was successfully inserted across the necks of the 2 aneurysms without complications. The post-procedure angiogram showed obvious contrast retention in the large aneurysm sac, while no contrast was seen in the small **(**Fig. 1  B**)**. Also, post-procedure Vaso-CT revealed excellent apposition of the stents. The PLED bridged aneurysmal necks in both aneurysms completely **(**Fig. 1  C**)**. The patient was put on daily aspirin 75 mg and clopidogrel 75 mg. She was discharged home 5 days after the endovascular procedure.

Ten days after discharge from the hospital, the patient was re-admitted at our institution again because of proptosis and conjunctival congestion. A repeated cerebral angiography revealed CCF triggered by delayed CCA rupture. The venous drainage of the CCF were as follows: anteriorly via the sphenoparietal sinus (SS) or cortical vein and superior ophthalmic vein (SOV), inferiorly via the pterygoid plexus (PP), inferior petrosal sinuses (IPS) and superior petrosal sinuses (SPS) **(**Fig. 1  D and E).

After careful evaluation of the CCF and the implanted PLED, the utilization of coils and/or ONYX (ev3, Irvine, CA) to occlude the fistula via the trans-arterial approach was not possible. Therefore, we adopted the trans-venous approach. We advanced the microcatheter to the origin of right SOV (Fig. 1  F) and diffused ONYX into it through ipsilateral IPS and CS. After eliminating the SOV drainage, we further diffused ONYX in order to eliminate the venous drainage in other directions. When we observed little drainage at SS and PP (Fig. 1  G and H), we stopped the diffusing of ONYX because the flow and speed of residual venous drainage were very small and slow.

Prior to his hospital discharge, a DSA was done to evaluate the residual cortical vein drainage. We observed a little decreased in blood flow in the cortical vein and a relieve of his symptomatology. Three months follow-up with DSA revealed occlusion of the CCF (Fig. 1  I and J). Two years follow reveal no further complications and the patient is well.

## 3 Discussion

Dual aneurysms arising from the ICA is a very rare occurrence. Clinoid segment aneurysms (CSAs) usually originates from the carotid dural rings^[1,3]^ while CCAs are often a direct communication between the ICA and the CS.^[1,3]^ Nevertheless, aneurysms at these segments often rupture resulting in high flow shunts between the ICA and the CS leading to CCF. Our case is a complex concomitant occurrence of a CSA and a CCA complicated DAR resulting in CCF after our initial treatment of the patient with PLED.

The clinoidal segment of the ICA is situated in between the CS and the subarachnoid space.^[4,18]^ This segment of the ICA is inadequately comprehended due to its complex and variable anatomy.^[1]^ The name clinoidal segment was arrived at because the ICA is link to the anterior clinoidal process (ACP).^[1,5]^ The ACP meticulously juxtaposed to the lateral wall of the ICA. This segment is just proximal to the usual location of the ophthalmic artery (OA) and superior hypophyseal artery (SHA).^[4]^ The OA derived is from the dorsomedial wall while the SHA is derived from the ventromedial wall.^[4]^

Fischer observed that, aneurysms originating from the third cervical vertebra(C3) segment of the clinoidal artery often enlarge via the distal dural ring leading to either optic nerve compression or SAH.^[19,20]^ Nevertheless, Bouthillier et al observed same symptoms for aneurysms originating from the fifth cervical vertebra(C5).^[21]^ The complex and variable anatomical location of CSA often makes endovascular treatment very challenging.^[20]^ Meyer et al observed a complication rate of 10% during their endovascular management of CSA.^[20]^ The risk of rupture of CSAs with resultant subarachnoid hemorrhage (SAH) depends on their anatomic connections with the upper dural ring.^[20]^ We utilized PLED in management of our CSA. During our follow-ups, this segment of aneurysm did not present with a complication because of the small size of the aneurysm.

On the other hand, CCAs constitutes about 2% to 9% of all aneurysms in the brain.^[8–10]^ The etiology of CCAs can be traumatic, infectious, or idiopathic.^[11]^ CCAs are often classified into high and low-risk aneurysm.^[10]^ The classification is based on precise anatomical location of the aneurysms. The potentials of rupture of CCAs as well as management options are founded on this classification system.^[10]^ CCAs are often asymptomatic and are detected incidentally during radiological evaluation. Nevertheless, symptomatic patients present with compressive symptoms on the 3rd, 4th, 5^th^, and 6th cranial nerves.^[8,11,22]^ Unruptured CCAs often present with diplopia as well as pain due to mass effect.^[10]^ Unilateral headaches, retro-orbital pain as well as facial pain are the most reported pain syndromes.^[8,9]^ Nevertheless, optic neuropathy, ocular sympathetic paresis, corneal hypesthesia, as well as trigeminal dysesthesias are most often also associated with CCAs.^[9,10]^ Our patient presented with diplopia as his principal complain.

DSA still remains the most valuable intracranial vascular imaging modality though the initial work out of their diagnosis usually starts with Magnetic resonance angiogram (MRA) and computed tomographic angiogram (CTA). Furthermore, 3D-DSA which is an advanced rotational cerebral angiography is very crucial in assessing specific anatomical classification as well as accurate image reconstructions of CCAs.^[9,10]^ MRI and CT-scan were very helpful during our initial work-out leading to the substantive diagnosis of both the CSA and CCAs.

The principal treatment options for CCAs is either surgery or endovascular techniques.^[10]^ Cure of CCAs often focus on either occlusion of the aneurysm sac or reconstruction of the aneurysmal walls.^[10]^ The most preferred treatment modality for small CCAs as well as those which ruptures into CS is endovascular coiling.^[23]^ Nevertheless, ICA occlusion is the most suitable treatment option for large and giant CCAs. In cases were ICA occlusion is not possible, selective coiling, bypass surgery prior to ICA occlusion, or conservative treatment are usually the possible alternatives.^[23]^

Occlusive approaches may involve surgical ligation of parent artery or via endovascular procedures.^[10,11]^ On the other hand, direct microsurgical clipping of the aneurysm usually constitutes the reconstruction.^[10,23]^ Moreover, reconstruction is often done via coil embolization with or without vascular reconstruction device, FDDs, or liquid embolic agents.^[10]^ We utilized PLED in the management of this segment of the aneurysm with DAR as the main complication which warranted a second endovascular procedure. Studies have confirmed that, CCAs can rupture resulting in CCF with occasionally SAH if the aneurysm extends through the dura or after endovascular procedures.^[13,16]^ Our follow-ups after the initial operation was to detected any associated complications.

DAR is one of the rare outcomes following the management of ICAs with FDDs.^[15,16]^ DAR or parent vessel injury can result in an anomalous connection between the ICA and the CS, initiating a CCF.^[15,16]^ The cardinal presentation in our patient was proptosis and conjunctival congestion. This symptomatology prompted us on a complication as a result of our management.

MRI and CT-scan are very valuable in determining the accompanying cerebral parenchymal injury, edema, as well as orbital anomalies.^[24]^ Orbital anomalies such as proptosis, distended ophthalmic veins, protruding CS, as well as thickness of the extraocular muscles.^[24]^ Furthermore, MRI is very accurate in detecting intracerebral hemorrhage, ischemia as well as incidental injuries to the cranial nerves.^[24]^

Nevertheless, DSA is the gold standard imaging modality use in assessing and detecting CCF.^[13,15]^ DSA is also very virtual in guiding endovascular management.^[14]^ We utilized DSA to assess the size and location of the fistula in our case. This imaging modality also aided in classifying the fistula as to direct or indirect.^[24]^ We also identified the outflow pathway from the CS, CS varix as well as high-risk hemodynamic anomalies like cortical venous drainage.

Endovascular management of CCFs stereotypically involves either trans-arterial obliteration of the fistulous site or trans-venous embolization via the CS.^[15,25]^ The gold standard treatment modality for CCFs is usually trans-arterial embolization.^[13]^ We successfully occluded the fistula with ONXY embolization agent via the trans-venous approach. We adopted the trans-venous approach because PLEDs where already inserted into both aneurysms via the artery route. Nevertheless, complications like thromboembolism as well as ischemic events are often associated with endovascular management of CCF.^[13]^ Other complications like edema, ocular anomalies, pseudoaneurysm formation, as well as changes in arterial flow leading to fetal hemorrhage has also been reported.^[13,26]^ Two years follow-ups of the patient revealed no such reported complication above.

## 4 Conclusion

A complex concomitant occurrence of CSA and CCA complicated with a delay rupture of the CCA resulting in CCF after treatment with PLED is a very rare occurrence. PLED was the best endovascular treatment option though DAR was inevitable. Although the trans-arterial approach may be the gold standard treatment modality for managing CCF, the complex nature of our case made us opt for trans-venous approach. The trans-venous route is very appropriate for fistulas with complex parent arteries.

## Author contributions

**Conceptualization:** Wang Ting, Seidu A Richard, Zhang Changwei, Wang Chaohua, Xie Xiaodong.

**Data curation:** Wang Ting, Seidu A Richard, Zhang Changwei, Wang Chaohua, Xie Xiaodong.

**Formal analysis:** Wang Ting, Seidu A Richard, Zhang Changwei, Wang Chaohua, Xie Xiaodong.

**Investigation:** Wang Chaohua, Xie Xiaodong.

**Methodology:** Seidu A Richard, Zhang Changwei.

**Resources:** Xie Xiaodong.

**Supervision:** Zhang Changwei, Wang Chaohua.

**Writing – original draft:** Seidu A Richard.

**Writing – review & editing:** Wang Ting, Seidu A Richard, Zhang Changwei, Wang Chaohua, Xie Xiaodong.
